# Integrated analysis on the N6‐methyladenosine‐related long noncoding RNAs prognostic signature, immune checkpoints, and immune cell infiltration in clear cell renal cell carcinoma

**DOI:** 10.1002/iid3.513

**Published:** 2021-08-25

**Authors:** Yuqin Qiu, Xiaogang Wang, Zhenjia Fan, Shanhui Zhan, Xin Jiang, Jinchang Huang

**Affiliations:** ^1^ Third Affiliated Hospital Beijing University of Chinese Medicine Beijing China; ^2^ Department of Emergency Medicine, Beijing Haidian Hospital Haidian Section of Peking University Third Hospital Beijing China; ^3^ Institute of Acupuncture and Moxibustion in Cancer Care Beijing University of Chinese Medicine Beijing China

**Keywords:** clear cell renal cell carcinoma, immune cell infiltration, immune checkpoint, long noncoding RNA, N6‐methyladenosine, prognosis

## Abstract

**Background:**

Patients with advanced clear cell renal cell carcinoma (ccRCC) have a poor prognosis and lack effective prognostic biomarkers. N6‐methyladenosine‐related lncRNAs (m6A‐related long noncoding RNAs [lncRNAs]) have been confirmed to be associated with the development of multiple tumors, but its role in ccRCC is not clear.

**Methods:**

Gene expression data and clinical information of ccRCC patients were extracted from The Cancer Genome Atlas Database. The prognostic m6A‐related lncRNAs were obtained by Pearson's correlation analysis and univariate Cox regression analysis. Afterward, the cluster classification and its correlation with prognosis, clinical characteristics, and immunity were analyzed. LASSO regression was used to establish the prognostic risk model. The predictive performance of the prognostic model was evaluated and validated by survival analysis and receiver operating characteristic curve analysis, et al. The expression of immune checkpoints and immune cell infiltration in patients with different risks were systematically analyzed.

**Results:**

A total of 27 prognostic m6A‐related lncRNAs were identified. These m6A‐related lncRNAs were differentially expressed between tumor and normal tissues. Among them, 24 high‐risk m6A‐related lncRNAs were overexpressed in Cluster 2 and correlated with poor prognosis, low stromal score, high expression of immune checkpoints, and immunosuppressive cells infiltration. Based upon, a prognostic risk model composed of seven m6A‐related lncRNAs was constructed. After a series of analyses, it was proved that this model had good sensitivity and specificity, and could predict the prognosis of patients with different clinical stratification. The expression of *PD‐1, PD‐L1, CTLA‐4, LAG‐3, TIM‐3*, and *TIGIT* were significantly increased in the high‐risk patients, and there was a correlation between the risk score and immune cell infiltration.

**Conclusions:**

The seven m6A‐related lncRNAs prognostic risk signature showed reliable prognostic predictive power for ccRCC and was associated with the expression of immune checkpoints and immune cell infiltration. This seven m6A‐related lncRNAs signature will be helpful in managing ccRCC and guiding individualized immunotherapy.

## Graphical abstract

M6A‐related long noncoding RNAs can well predict the prognosis of clear cell renal cell carcinoma of different ages, genders, pathological characteristics and stages and disease progression, and are associated with the expression of immune checkpoints and immune cell infiltration.

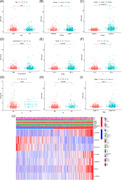

## 1 INTRODUCTION

Renal cell carcinoma (RCC) is a common genitourinary tumor, accounting for 3%–5% of all adult malignancies. Its incidence is increasing in recent years, and it occurs more in males than in females.^1^ Clear cell renal cell carcinoma (ccRCC) is the most common pathological type of RCC, accounting for approximately 75% of RCCs.^2^ The classical RCC triad is hematuria, flank pain, and abdominal masses. However, most RCC patients are asymptomatic at onset and are already at an advanced stage at the time of diagnosis. Although the 5‐year survival rate of early RCC is 93%, the prognosis of patients with locally advanced or metastatic RCC is generally poor, with a 5‐year survival rate of only approximately 12%.^3^, ^4^ Therefore, an effective prognostic biomarker is of great significance for early diagnosis and treatment as well as for improving patient prognosis. ccRCC is resistant to traditional radiotherapy and chemotherapy, and the treatment modalities for patients with advanced ccRCC are very limited. Targeted drugs such as sorafenib are the standard means of treatment for the advanced‐stage patients. In recent years, some clinical studies have demonstrated that immunotherapy is effective for advanced RCC.^5^, ^6^ As the awareness of tumor immunity continues to rise, novel immunotherapeutic drugs continue to emerge. Therefore, novel biomarkers are critical for the better selection of patients who may benefit most from these treatments.^7^

Long noncoding RNAs (lncRNAs) are a group of noncoding RNAs with a length greater than 200 nucleotides.^8^ They can regulate gene expression through epigenetic regulation, transcriptional regulation, and posttranscriptional regulation and are involved in various biological processes such as cell proliferation, differentiation, apoptosis, and migration, as well as regulation of tumor cell cycle.^9^, ^10^, ^11^ An increasing number of lncRNAs have been shown to be abnormally expressed in cancers and have a significant correlation with treatment and clinical prognosis, indicating the possibility of using lncRNAs as novel tumor biomarkers and therapeutic targets.^12^ For example, the level of lncRNA *small nucleolar RNA host gene 14 (SNHG14)* is significantly increased in ovarian cancer tissues, and the inhibition of *SNHG14* significantly inhibits the migration and invasion of cells.^13^ The upregulation of lncRNA *gastric cancer metastasis‐associated long noncoding RNA* in gastric cancer tissues is associated with metastasis in patients.^14^ The lncRNA *urothelial cancer‐associated 1 (UCA1)* is significantly upregulated in RCCs and is positively correlated with tumor differentiation and tumor node metastasis (TNM) staging. *UCA1* promotes the malignant phenotype of RCCs by regulating of the miR‐182‐5p/DLL4 axis.^15^

N6‐methyladenosine (m6A) methylation occurs in the N6‐position of adenosine.^16^ It was first discovered in eukaryotic messenger RNA (mRNA) in 1970 and is considered to be the most common RNA modification.^17^ M6A modification is a reversible and dynamic process. It is regulated by three types of m6A regulators. M6A RNA modification is catalyzed by methyltransferases (called the “writers,” such as *METTL3, METTL14*, and *RBM15*); removed by demethylases (called the “erasers,” such as *FTO* and *ALKBH5*); and recognized by binding proteins (called the “readers,” such as *YTHDC1‐2 and YTHDF1‐3*).^16^, ^17^ M6A modification has been demonstrated to be involved in the regulation of the occurrence and progression of a variety of cancers, including ccRCC and breast cancer.^18^, ^19^ Studies have shown that lncRNAs are widely modified by m6A^20^ and that the interaction between the two is involved in tumor progression, metastasis, drug resistance, and immune responses.^21^ Studies have revealed that m6A‐related lncRNAs can be used as potential biomarkers to predict the prognosis of patients with glioma.^22^ However, the biological role of the interaction between lncRNA expression and m6A‐related genes in ccRCC has not been explored.

In this study, we used bioinformatics to screen for m6A‐related lncRNAs that are associated with the prognosis of ccRCC and constructed a prognostic model. This prognostic model will help to assess the prognosis of ccRCC patients and guide clinical treatment. Our results will provide a basis for further exploration of the potential mechanism of m6A modification of lncRNAs in ccRCC.

## 2 MATERIALS AND METHODS

### 2.1 Data acquisition and collection

Transcriptomic sequencing (RNA‐seq) data, fragments per kilobase of transcript per million mapped reads, and related clinical information of ccRCC patients were downloaded from The Cancer Genome Atlas (TCGA) database (https://portal.gdc.cancer.gov/, until February 1, 2021). The Genome Reference Consortium Human Build 38 (GRCh38) was downloaded from GENCODE (https://www.gencodegenes.org/human), and the expression data of lncRNAs in the transcriptome were extracted based on the gene biotype. These data were obtained from a public database, and therefore, ethics approval was not required.

### 2.2 Extraction of m6A‐related lncRNAs associated with prognosis

According to previously published studies, we found 23 m6A‐related genes, namely, methyltransferases *METTL3, METTL14, METTL16, WTAP, VIRMA, ZC3H13, RBM15*, and *RBM15B*; demethylases *ALKBH5* and *FTO;* and recognition proteins *YTHDC1. YTHDC2, YTHDF1, YTHDF2, YTHDF3, HNRNPC, FMR1, LRPPRC, HNRNPA2B1, IGFBP1, IGFBP2, IGFBP3*, and *RBMX*.^23^, ^24^ The expression matrix of the m6A‐related genes in ccRCC samples was extracted from the TCGA transcriptome data. m6A‐related lncRNAs were obtained by coexpression analysis of m6A‐related genes and lncRNAs by the Pearson correlation coefficient. The filtered correlation coefficient was more than 0.7; *p* < .001. These lncRNAs were combined with clinical survival data, and univariate Cox regression analysis was performed with R software version 4.0.3 survival package to identify the m6A‐related lncRNAs associated with ccRCC prognosis (*p* < .001).

### 2.3 Biological characteristics of m6A‐related lncRNAs

To investigate the clinical significance of m6A‐related lncRNAs that are associated with prognosis, we used the Consensus Clustering algorithm and the ConsensusClusterPlus package^25^ of R to classify ccRCC patients into different subtypes based on the expression of prognosis‐related lncRNAs. The differences in clinical features and prognosis were analyzed. Consensus clustering is a method that provides quantitative evidence for determining the membership and number of possible clusters in a data set and is widely used in cancer genomics. Because resampling was used, the obtained clustering results had excellent stability.

To further explore the biological processes mediated by m6A‐related lncRNAs, we used GSEA software (version 4.1.0) to determine gene expression enrichment for different subtypes in the Molecular Signatures Database Collection (c5.go.bp.v7.2.symbols.gmt; c2.cp.kegg. v7.2symbols.gmt) to analyze the difference of Gene Ontology (GO) functional enrichment and Kyoto Encyclopedia of Genes and Genomes (KEGG) pathway enrichment. A normalized enrichment score (NES) > 1 and nominal *p* value (NOM *p*‐val) < .05 were used to determine the difference between different genotypes.

### 2.4 Correlation analysis of m6A‐related lncRNAs with tumor microenvironment and immune cell infiltration

LncRNAs are involved in the regulation of the tumor microenvironment (TME) and signaling transduction in tumor cells. To understand the correlation between m6A‐related lncRNAs and TME, we used the ESTIMATE algorithm in the R estimate package^26^ to calculate the ratio of immune cells and stromal cells in the TME for each sample. The LIMMA package was used for difference analysis and the ggpubr package was used to visualize the results. Then, we used the CIBERSORT algorithm^27^ to evaluate the immune cell infiltration of different clusters. CIBERSORT is a tool for the deconvolution of the expression matrix of human immune cell subtypes based on the principle of linear support vector regression. Immune cell infiltration can be estimated by RNA‐seq data. Monte Carlo sampling methods were used to calculate the empirical *p* value of deconvolution to represent the accuracy of the results. *p* < .05 indicated that the inferred cell composition was reliable. Leukocyte signature matrix (LM22) contains 547 genes that distinguish 22 human immune cell phenotypes, including seven T‐cell types, naive and memory B cells, plasma cells, natural killer (NK) cells, and myeloid subsets. We used the CIBERSORT algorithm to analyze the RNA‐seq expression profile based on the LM22 classification. We evaluated the abundance of immune cell subsets in samples and visualized the differences in immune cell infiltration between different types.

### 2.5 Correlation analysis of m6A‐related lncRNAs and immune‐related genes

To explore the correlation between m6A‐related lncRNAs and immune‐related genes, we selected programmed cell death 1 *(PD‐1)*, programmed cell death‐ligand 1 *(PD‐L1)*, cytotoxic T lymphocyte‐associated antigen 4 *(CTLA‐4)*, lymphocyte activation gene‐3 *(LAG‐3)*, T‐cell immunoglobulin, and mucin domain‐containing protein 3 *(TIM‐3)* and T‐cell immunoglobulin and ITIM domain (*TIGIT)*, that are the key genes associated with currently used tumor immune checkpoint inhibitors, and we used the R corrplot package^28^ to plot the correlation analysis between the 27 m6A‐related lncRNAs and these genes using the function cor.mtest to obtain the *p* values. The differential expression of the above genes between tumor and normal samples and different clusters was analyzed using the limma package^29^ of R software and Wilcoxon test (*p* < .05).

### 2.6 Construction and validation of the prognostic risk model

First, the R caret package^30^ was used to randomly divide the samples with complete survival information in the TCGA database into two groups, that is, a training set and a test set, with each set consisting of approximately 50% of cases. Subsequently, LASSO regression analysis was used to construct the prognostic model. The optimized model was obtained using the penalty parameter estimated by 10‐fold cross‐validation. The risk score of the prognostic model = $\sum_{i=1}^{n}\left(\mathrm{Coefi}\times \beta i\right)$, where *Coef* represents the regression coefficient, and *β* represents the m6A‐related lncRNA expression value. The risk score for each patient was calculated using this formula, and then, the training set and test set were divided into a high‐risk group and a low‐risk group based on the median score. The R survival package was used for Kaplan–Meier analysis. The log‐rank test was used to compare the overall survival rates for the high‐ and low‐risk groups. A time‐dependent receiver operating characteristic (ROC) curve was plotted. The area under the curve (AUC) > 0.60 was considered an acceptable prediction. In addition, we also used univariate and multivariate Cox regression analyses to assess whether the risk score could be an independent prognostic factor for ccRCC. To evaluate the predictive ability of the model for different populations, Kaplan–Meier analysis was performed for age, sex, grade, stage, and TNM staging. Finally, we analyzed the correlation between risk scores and clinicopathological features to assess the ability of this model to predict ccRCC progression.

### 2.7 Analysis of immune checkpoints and tumor‐infiltrating immune cells in patients with different prognostic risks

To understand the significance of this risk model in the assessment of the immune microenvironment and immunotherapeutic efficacy in ccRCC, we analyzed the differences in the expression of immune checkpoints between the high‐ and low‐risk patients and the correlation between risk scores and tumor‐infiltrating immune cells.

## 3 RESULTS

### 3.1 Identification of m6A‐related lncRNAs associated with ccRCC prognosis

**Figure 1:**
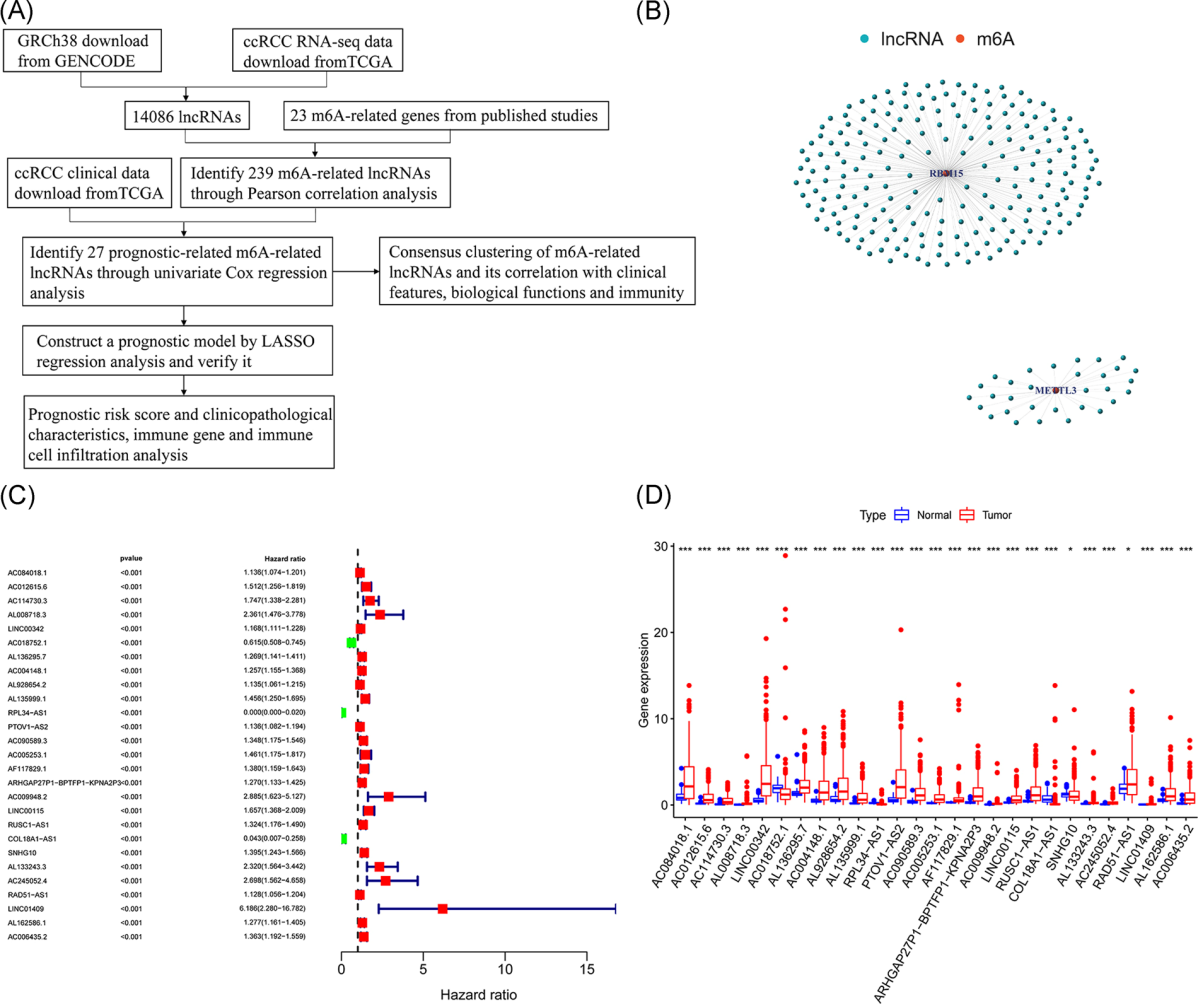
Identification of m6A‐related lncRNAs associated with ccRCC prognosis. (A) Study flow chart. (B) Coexpression map of m6A and lncRNAs. (C) The forest plot of screening prognostic m6A‐related lncRNAs by univariate Cox regression analysis. (D) The box plot of the expression of 27 m6A‐related lncRNAs in tumors and normal tissues. ccRCC, clear cell renal cell carcinoma; lncRNA, long noncoding RNA; TCGA, The Cancer Genome Atlas. *p < .05, **p < .01, and ***p < .001

The RNA‐seq data of 611 samples (tumor samples, 539; normal samples, 72) downloaded from TCGA were combined with GRCh38 downloaded from GENCODE to obtain 14086 lncRNAs. The workflow was shown in Figure 1A. Pearson correlation analysis of these lncRNAs and the expression matrix of m6A‐related genes yielded a total of 239 lncRNAs (|Pearson *R* | > .7; *p* < .001) that were positively correlated with *RBM15* and *METTL3* (Figure 1B and Table S1). Through univariate Cox regression analysis, 27 m6A‐related lncRNAs that were correlated with prognosis were obtained (*p* < .001; Figure 1C and Table S2).

The differential expression of these lncRNAs between tumor and normal tissues was analyzed using the limma package of R software and Mann–Whitney Wilcoxon test (*p* < .05). Three low‐risk lncRNAs, *AC018752.1, RPL34‐AS1*, and *COL18A1‐AS1*, had higher expression in normal tissue than in tumor tissue, and the remaining 24 high‐risk lncRNAs (*AC084018.1, AC012615.6, AC114730.3, AL008718.3, LINC00342, AL136295.7, AC004148.1, AL928654.2, AL135999.1, PTOV1‐AS2, AC090589.3, AC005253.1, AF117829.1, ARHGAP27P1‐BPTFP1‐KPNA2P3, AC009948.2, LINC00115, RUSC1‐AS1, SNHG10, AL133243.3, AC245052.4, RAD51‐AS1, LINC01409, AL162586.1*, and *AC006435.2*) were significantly overexpressed in tumor tissues (Figure 1D).

### 3.2 Consensus clustering of m6A‐related lncRNAs correlates with prognosis and biological functions in ccRCC

**Figure 2:**
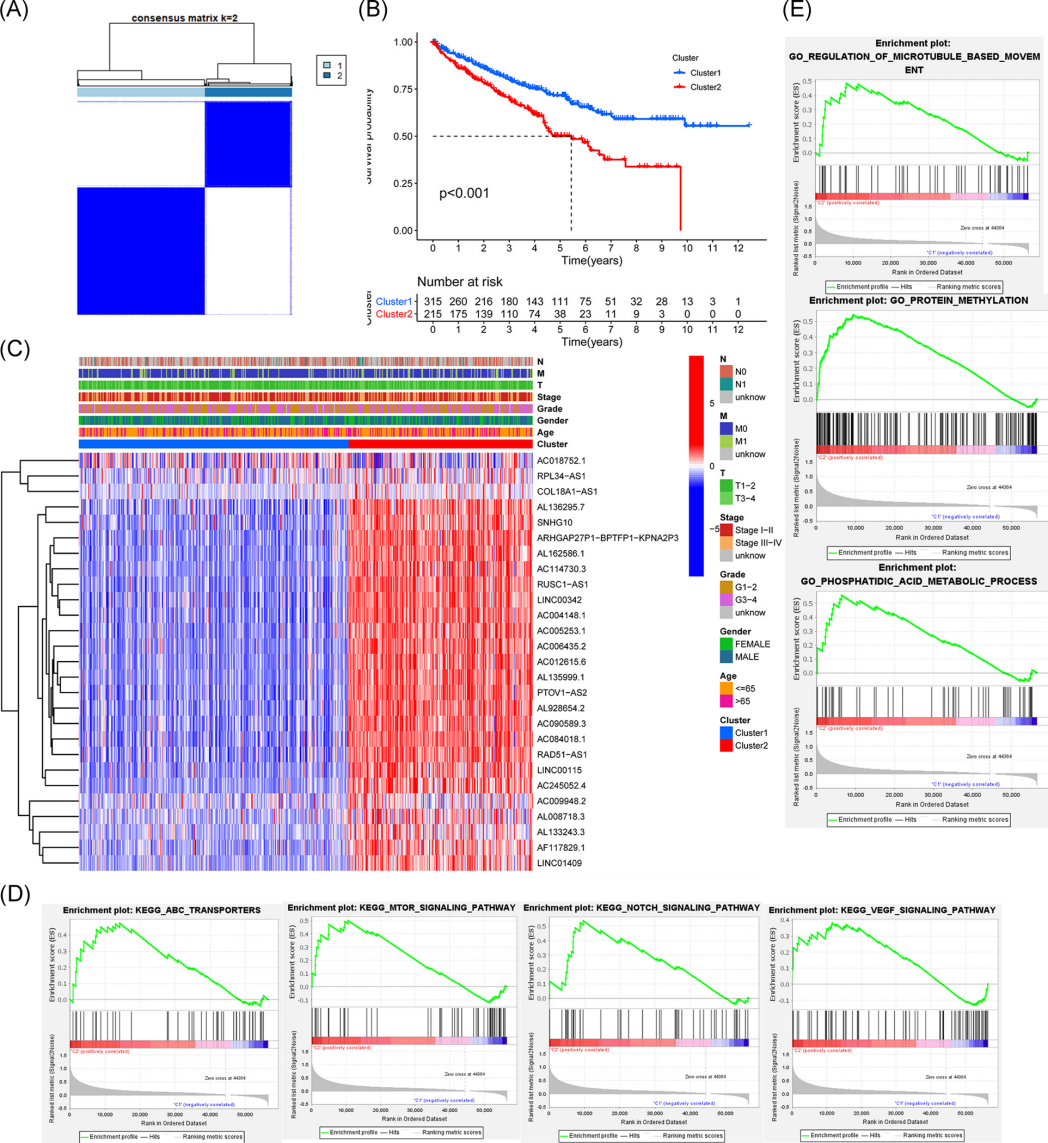
Consensus clustering of m6A‐related lncRNAs correlates with clinicopathological features and biological functions in ccRCC. (A) Consensus clustering matrix for *k* = 2. (B) Kaplan–Meier curves of overall survival for ccRCC patients in two clusters. (C) The heatmap of clinicopathologic features of the two clusters. (D) KEGG results showing the mTOR signaling pathway, ABC transporter, Notch signaling pathway, and VEGF signaling pathway are significantly enriched in Cluster 2. (E) GO results showing the biological processes related to protein methylation, regulation of microtubule‐based movement, and phospholipid acid metabolism are significantly enriched in Cluster 2. ABC, ATP‐binding cassette; ccRCC, clear cell renal cell carcinoma; GO, Gene Ontology; KEGG, Kyoto Encyclopedia of Genes and Genomes; lncRNA, long noncoding RNA; mTOR, mammalian target of rapamycin; VEGF, vascular endothelial growth factor

We adopted the resampling method to sample 80% of the samples and used the *K* means clustering algorithm to select the *k* value with the highest intracluster correlation, that is, *k* = 2, as the optimal number of clusters, based on the results of *k* typing from 2 to 9 (Figure S1A–C). A total of 530 ccRCC patients were classified into two subtypes, namely, Cluster 1 (*n* = 315) and Custer 2 (*n* = 215; Figure 2A). Kaplan–Meier analysis was performed with the R survival package to compare the prognosis of Cluster 1 and Cluster 2 by log‐rank test. It was found that the survival rate of Cluster 2 was significantly lower than that of Cluster 1 (*p* < .001; Figure 2B). Twenty‐four high‐risk m6A‐related lncRNAs were significantly overexpressed in Cluster 2, and there were no significant differences in age, sex, grade, stage, or TNM staging between the two clusters by *χ* ^2^ test (Figure 2C).

To evaluate the potential biological functions of m6A‐related lncRNAs, GO enrichment analysis and KEGG pathway enrichment analysis were conducted. The KEGG results showed that the mammalian target of rapamycin (mTOR) signaling pathway (NES = 1.81, NOM *p* < .012), ATP‐binding cassette (ABC) transporter (NES = 1.76, NOM *p* < .011), Notch signaling pathway (NES = 1.74, NOM *p* < .039), and vascular endothelial growth factor (VEGF) signaling pathway (NES = 1.62, and NOM *p* < .024) were significantly enriched in Cluster 2 (Figure 2D). GO enrichment results showed that biological processes related to protein methylation (NES = 2.17, NOM *p* < .002), regulation of microtubule‐based movement (NES = 2.05, NOM *p* < .001) and phospholipid acid metabolism (NES = 2.34, NOM *p* < .001) were significantly enriched in Cluster 2 (Figure 2E).

### 3.3 Consensus clustering of m6A‐related lncRNAs correlates with immune cell infiltration

**Figure 3:**
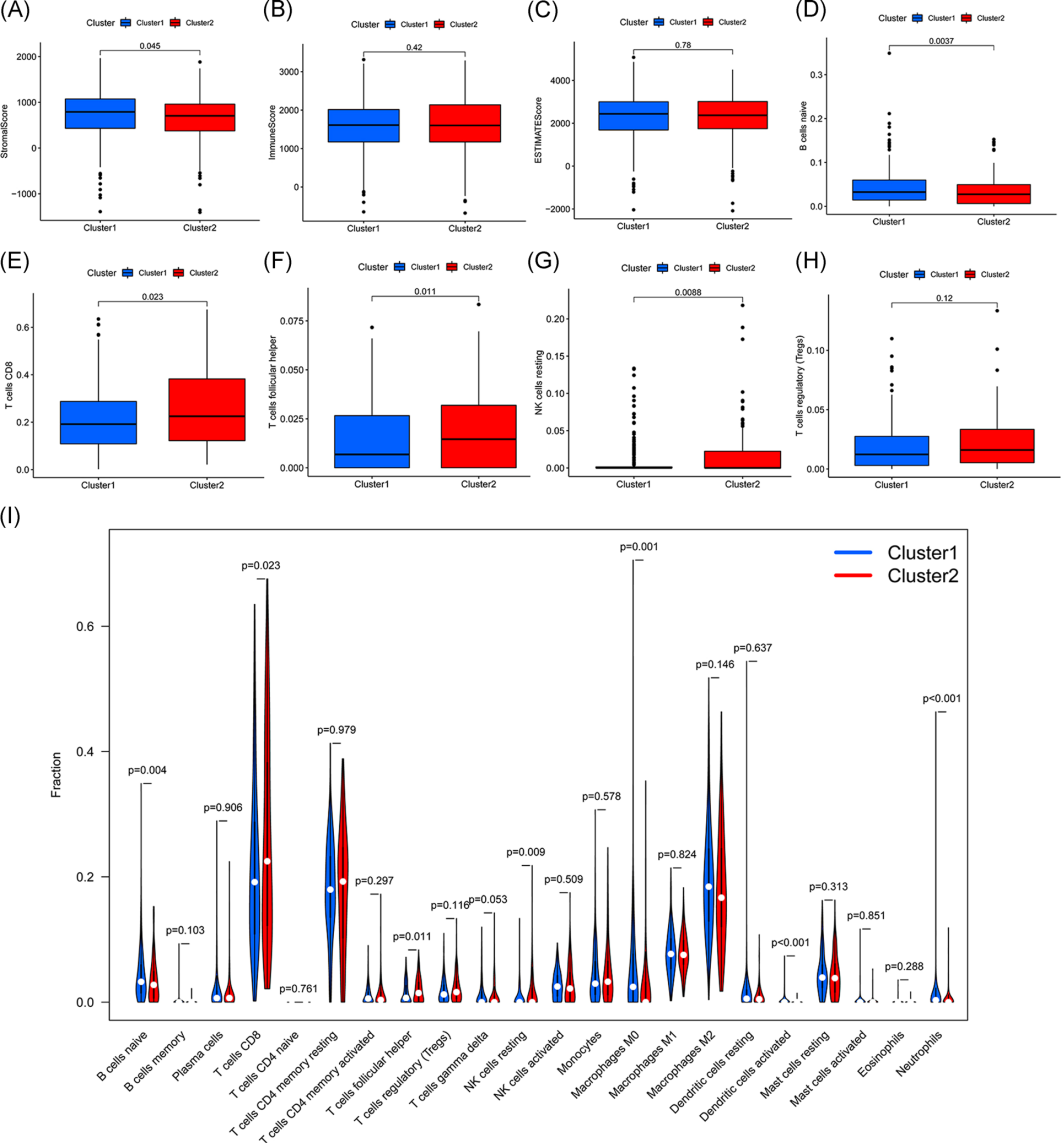
Association of the tumor microenvironment and immune cell infiltration with m6A‐related lncRNAs in ccRCC. (A) Stromal score, (B) immune score, and (C) ESTIMATE score of Clusters 1 and 2. (D–H) Differences in immune cell infiltration between Clusters 1 and 2. (I) The infiltration of 22 immune cell types in Clusters 1 and 2. ccRCC, clear cell renal cell carcinoma; lncRNA, long noncoding RNA

We predicted the content of stromal cells and immune cells by calculating the stromal scores and immune scores. The higher the score, the greater the proportion of the corresponding components. Finally, the tumor purity of each tumor sample was calculated based on the sum of the 2 (ESTIMATE score); the higher the score, the lower the tumor purity. It was found that the stromal score of Cluster 1 was higher than that of Cluster 2 (*p* = .045; Figure 3A), indicating that the number of stromal cells in Cluster 1 was higher. However, there were no significant differences in the percentage of immune cells and tumor purity between the two clusters (Figure 3B,C).

In addition, we analyzed the infiltration of 22 types of immune cells in these two clusters (Figure 3I) and found that the infiltration of naive B cells in Cluster 1 was high (Figure 3D), while the infiltration of CD8 T cells, follicular helper T cells and resting NK cells in Cluster 2 was more prominent (Figure 3E–G). In addition, more immunosuppressive T regulatory cells (Tregs) were infiltrated in Cluster 2, although there was no statistical difference between the two clusters (Figure 3H).

### 3.4 Association of immune‐related genes with m6A‐related lncRNAs

**Figure 4:**
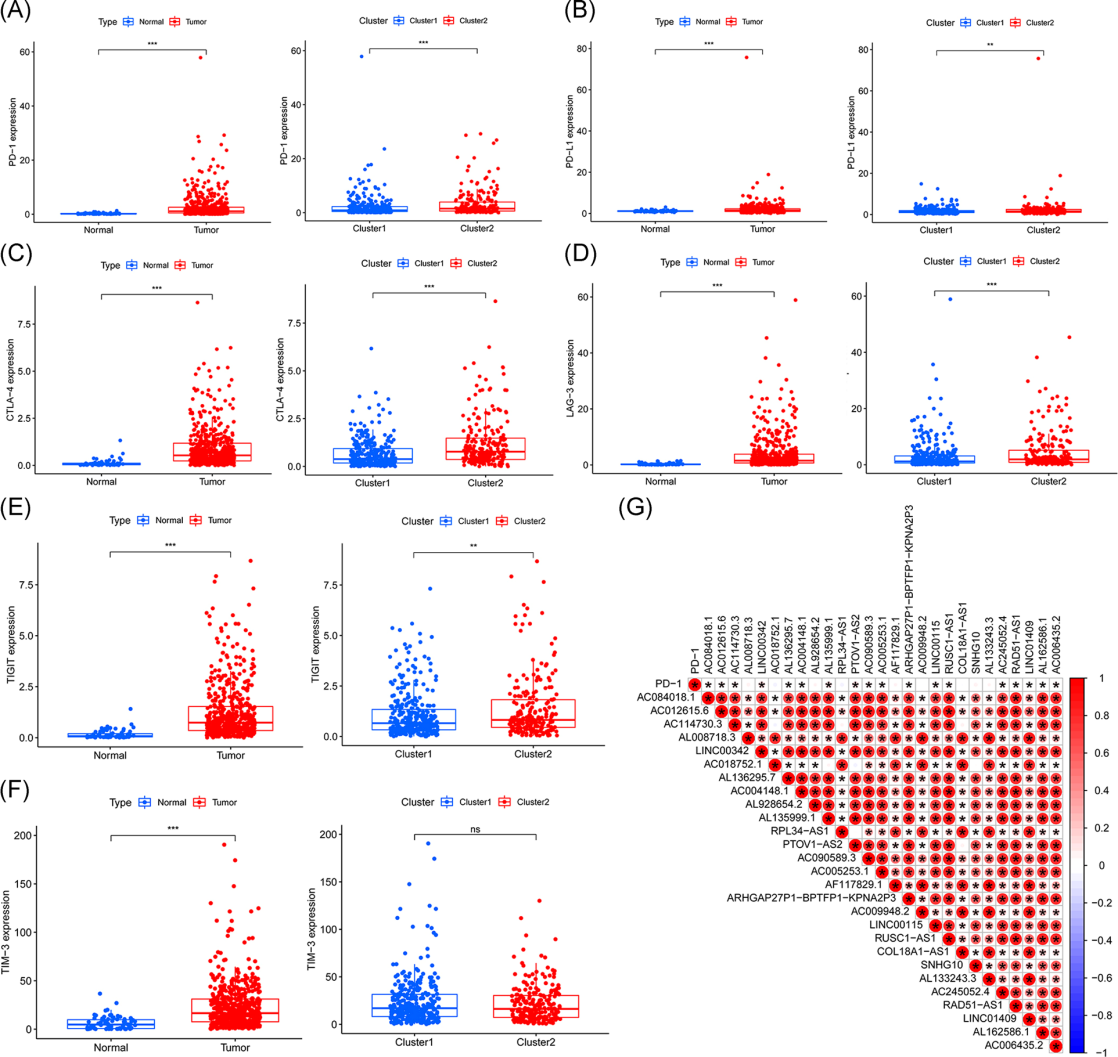
Association of immune genes with m6A‐related lncRNAs in ccRCC. The expression of (A) *PD‐1*, (B) *PD‐L1*, (C) *CTLA‐4*, (D) *LAG‐3*, (E) *TIGIT*, and (F) *TIM‐3* between normal and ccRCC tumor tissues and Clusters 1 and 2. *p < .05, **p < .01, and ***p < .001. (G) Correlation analysis plot of *PD‐1* and prognostic m6A‐related lncRNAs. Red represents a positive correlation, while blue represents a negative correlation, with darker colors indicating greater correlation coefficients. *Statistical significance between two genes, that is, *p* < .05. The blank space indicates no correlation between genes. ccRCC, clear cell renal cell carcinoma; lncRNA, long noncoding RNA

To explore the association between m6A‐related lncRNAs and immune‐related genes, we evaluated the differential expression of six types of immune checkpoints between these two clusters, and the correlation between the immune checkpoints and m6A‐related lncRNAs. We found that the expression of *PD‐1, PD‐L1, CTLA‐4, LAG‐3, TIM‐3*, and *TIGIT* in tumor samples was significantly higher than those in normal samples (all *p* < .001) and the expression of *PD‐1, PD‐L1, and CTLA‐4, LAG‐3*, and *TIGIT* in Cluster 2 was significantly higher than those in Cluster 1 (Figure 4A–F). Gene correlation analysis revealed some correlation between immune‐related genes and m6A‐related lncRNAs (Figures 4G and S2A–E). The expression of *PD‐1* positively correlated with *AC084018.1, AC012615.6, AC114730.3, LINC00342, AL136295.7, AC004148.1, AL135999.1, PTOV1‐AS2, AC090589.3, AC005253.1, ARHGAP27P1‐BPTFP1‐KPNA2P3, LINC00115, RUSC1‐AS1, AC245052.4, RAD51‐AS1, LINC01409, AL162586.1*, and *AC006435.2* (*p* < .05; Figure 4G). To further explore the interaction between m6A‐related lncRNAs, we also analyzed the correlation among these 27 lncRNAs. The results showed that except *AC114730.3* was not correlated with *AC018752.1, RPL34‐AS1*, and *COL18A1‐AS1*; *AC018752.1* was not correlated with *AL135999.1, PTOV1‐AS2*, and *SNHG10*; PTOV1‐AS2 was not correlated with *RPL34‐AS1* and *COL18A1‐AS1*, all the other lncRNAs showed positive correlations to various degrees (Figure 4G).

### 3.5 Construction and validation of the prognostic risk model

**Figure 5:**
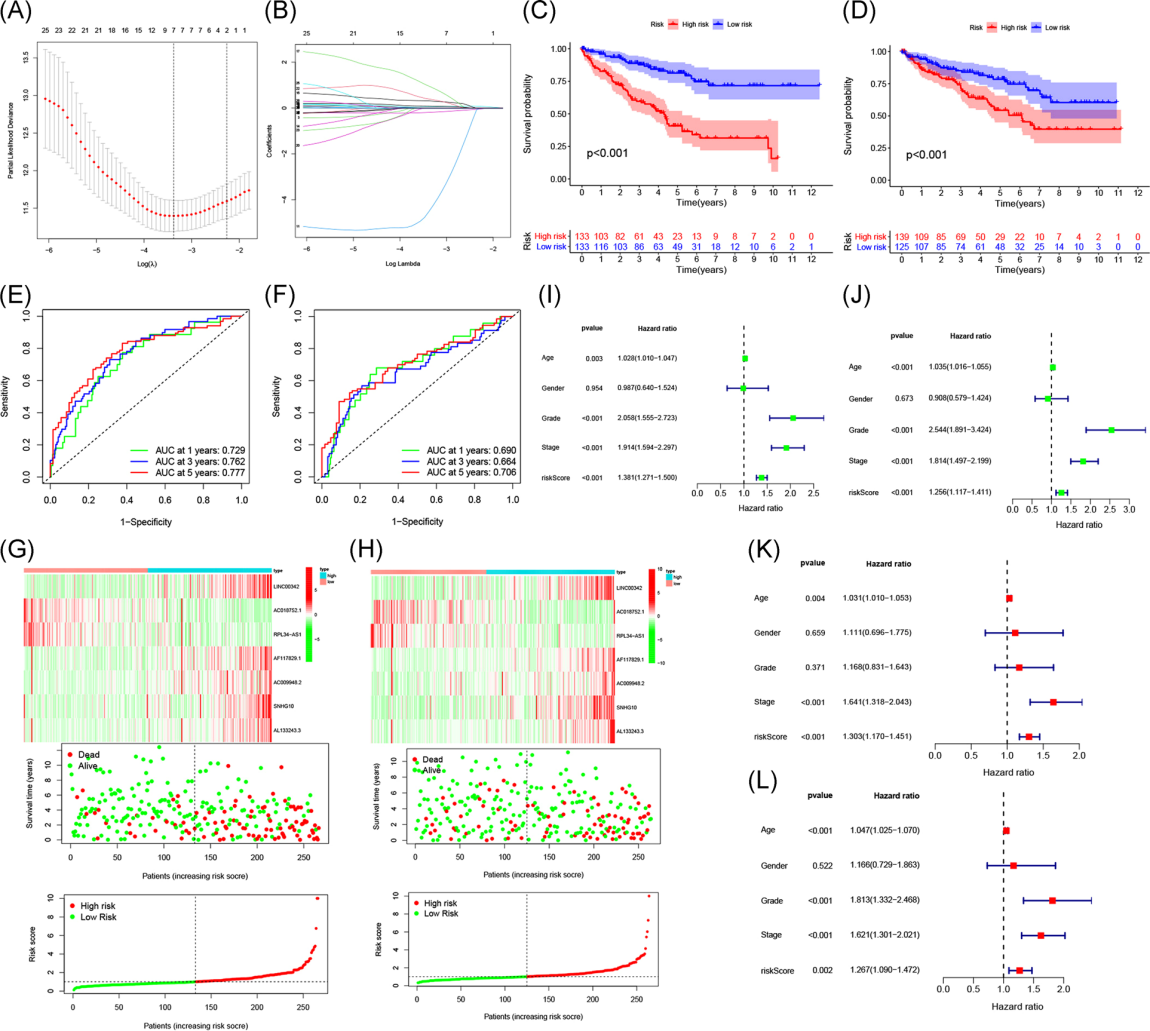
Construction and validation of the prognostic risk model. (A) Partial likelihood deviance for tuning the parameter selection in the LASSO regression model. (B) LASSO coefficient profiles of the 27 m6A‐related lncRNAs. (C, D) Kaplan–Meier curves and (E, F) ROC analysis of the prognostic model in the training set and test set. (G, H) Heat map, survival status, and risk score curve of ccRCC patients in the training set and test set. The (I, J) univariate and (K, L) multivariate Cox regression analysis evaluating the independent prognostic value of the risk score of the prediction model. ccRCC, clear cell renal cell carcinoma; lncRNA, long noncoding RNA; ROC, receiver operating characteristic

The ccRCC patients in the TCGA database were randomly divided into a training set (*n* = 266) and a test set (*n* = 264). There was no significant difference in the clinical baseline characteristics between the training set and the test set (*p* > .05; Table S3). LASSO regression analysis was performed for the prognostic m6A‐related lncRNAs (Figure 5A,B). Seven m6A‐related lncRNAs were obtained: *LINC00342, AC018752.1, RPL34‐AS1, AF117829.1, AC009948.2, SNHG10*, and *AL133243.3*. These seven lncRNAs were used to construct a prognostic model to obtain a risk score formula for each sample: risk score = (*LINC00342* × 0.0554) − (*AC018752.1* × 0.1872) − (*RPL34*‐*AS1* × 4.5020) + (*AF117829.1* × 0.3137) + (*AC009948.2* × 0.9556) + (SNHG10 × 0.0863) + (*AL133243.3* × 0.3944).

Based on the median risk score, patients in the training set were divided into high‐ and low‐risk groups for Kaplan–Meier survival analysis. The results showed that the overall survival of patients in the high‐risk group was lower than that of patients in the low‐risk group (*p* < .001; Figure 5C). The AUCs at 1, 3, and 5 years were 0.729, 0.762, and 0.777, respectively, indicating that this model could well predict the prognosis of patients (Figure 5E). To further verify the accuracy of the prognostic model, we used it to analyze the test set. The results showed that the survival rates for patients in the high‐risk and low‐risk groups were significantly different (*p* < .001; Figure 5D); the AUCs at 1, 3, and 5 years were 0.69, 0.664, and 0.706, respectively, suggesting that the prognostic model had excellent sensitivity and specificity (Figure 5F).

The risk score curve, survival status, and heat map of gene expression of patients in the high‐risk and low‐risk groups are shown in Figure 5G,H. As the risk score increased, the mortality of ccRCC patients increased. In high‐risk patients, five high‐risk lncRNAs (*LINC00342, AF117829.1, AC009948.2, SNHG10*, and *AL133243.3*) were upregulated, and two low‐risk lncRNAs (*AC018752.1* and *RPL34‐AS1*) were downregulated. In contrast, the expression of these lncRNAs in low‐risk patients were opposite.

Next, we performed univariate and multivariate Cox regression analyses to evaluate the prognostic value of risk scores and other clinical features of the prognostic model. Univariate Cox regression analysis indicated that age, grade, stage, and risk score were correlated with the prognosis of ccRCC patients (Figure 5I,J). Multivariate Cox regression analysis suggested that the age, stage, and risk score could be used as independent prognostic risk factors (Figure 5K,L).

### 3.6 Prognostic risk score correlates with clinicopathological characteristics

**Figure 6:**
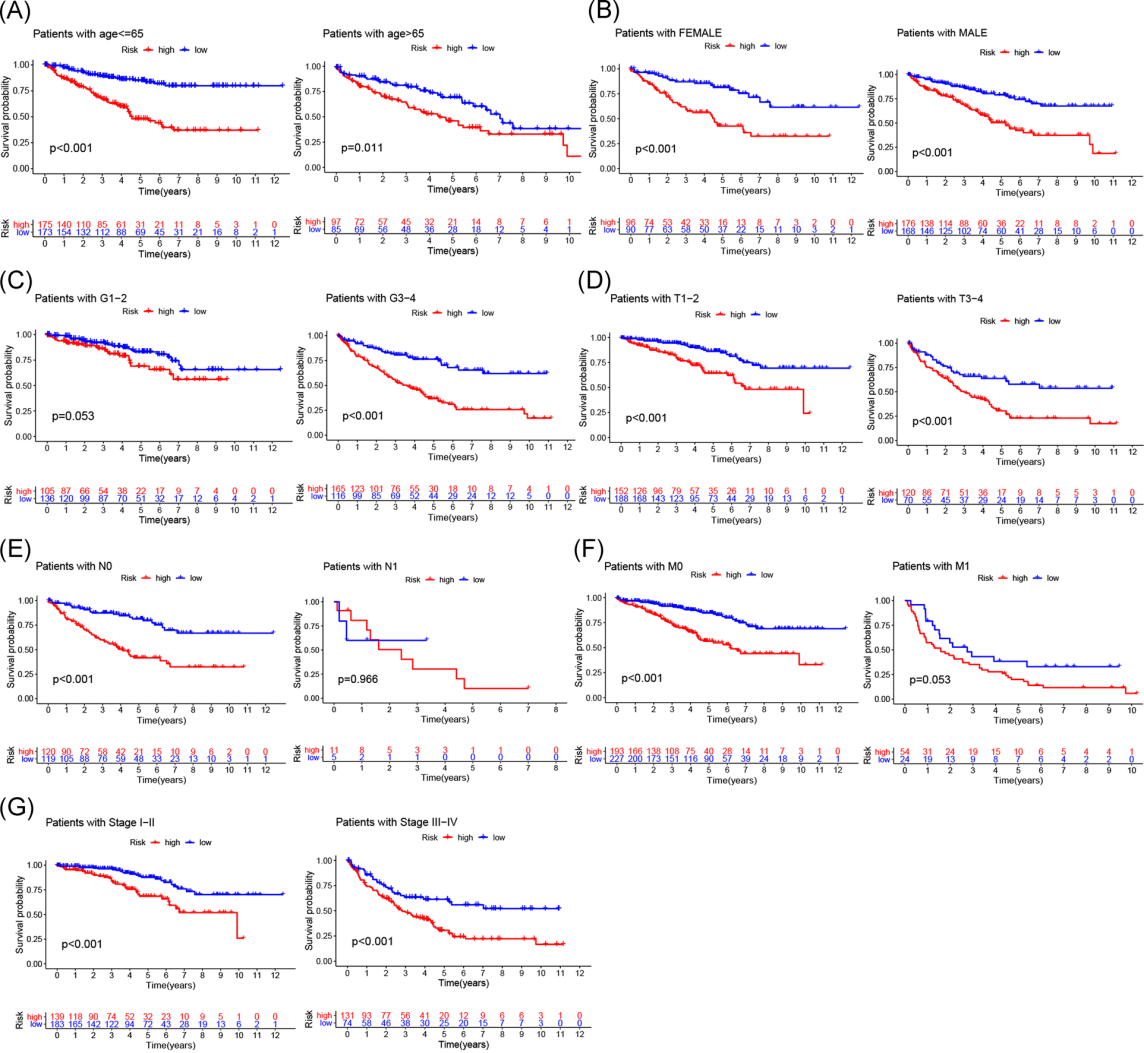
Kaplan–Meier survival analysis of the prognostic risk score for ccRCC stratified by clinicopathological characteristics. (A) age ≤ 65 and >65 years; (B) female and male; (C) patients with G1–2 and G3–4; (D) patients with T1–2 and T3–4; (E) patients with N0 and N1; (F) patients with M0 and M1; (G) patients with Stage I–II and III–IV. ccRCC, clear cell renal cell carcinoma

We further performed Kaplan–Meier survival analysis on different ages, sex, grades, stages, and TNM stages and compared the survival rates for high‐ and low‐risk patients in all stratifications to explore whether our prognostic model is suitable for patients with different clinical stratifications. The results are shown in Figure 6A–G. In older age (>65 years) or younger age (≤65 years) patients, males or females, with poorly differentiated or undifferentiated tumors (G3–4), different tumor sizes and invasion depths (T1–2, T3–4), no lymph node (N0) or distant metastasis (M0), and early‐stage (Stage I–II) or advanced stage (Stage III–IV) disease, the survival rate of high‐risk patients significantly decreased. These results indicated that the m6A‐related lncRNA‐based prognostic model is a powerful tool for predicting the prognosis of ccRCC patients with different clinical stratifications.

**Figure 7:**
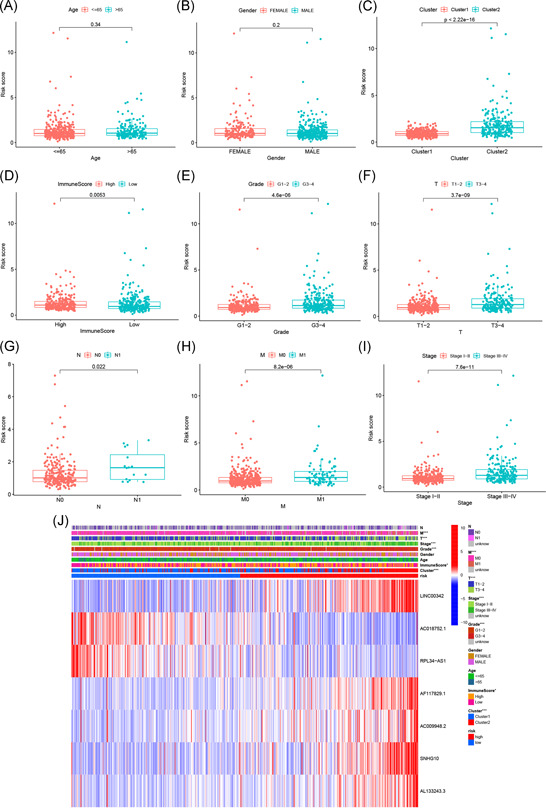
The correlation of risk score with the clinicopathological characteristics of ccRCC. (A) age ≤ 65 versus >65 years; *p* = .34; (B) male versus female; *p* = .2; (C) Cluster 1 versus Cluster 2; *p* < 2.22e−16; (D) high‐immune score versus low‐immune score; *p* = .0053; (E) G1–2 versus G3–4; *p* = 4.6e−06; (F) T1–2 versus T3–4; *p* = 3.7e–09; (G) N0 versus N1; *p* = .022; (H) M0 versus M1; *p* = 8.2e−06; (I) Stage I–II versus III–IV; *p* = 7.6e−11; (J) Heat map of risk scores and clinicopathological characteristics. ccRCC, clear cell renal cell carcinoma

Finally, we analyzed the correlation of risk scores with different clinicopathological characteristics and found that risk scores were significantly different among different clusters, grades, immune scores, TNM staging, and stage. Risk scores were significantly higher for patients in Cluster 2, G3–4, low‐immune scores, T3–4, N1, and M1 staging, and Stage III–IV (Figure 7A–J), suggesting that the expression of m6A‐related lncRNAs is associated with the occurrence and development of ccRCC and that the model is able to predict the progression of ccRCC.

### 3.7 Prognostic risk score correlates with the expression of immune checkpoints and immune cell infiltration

**Figure 8:**
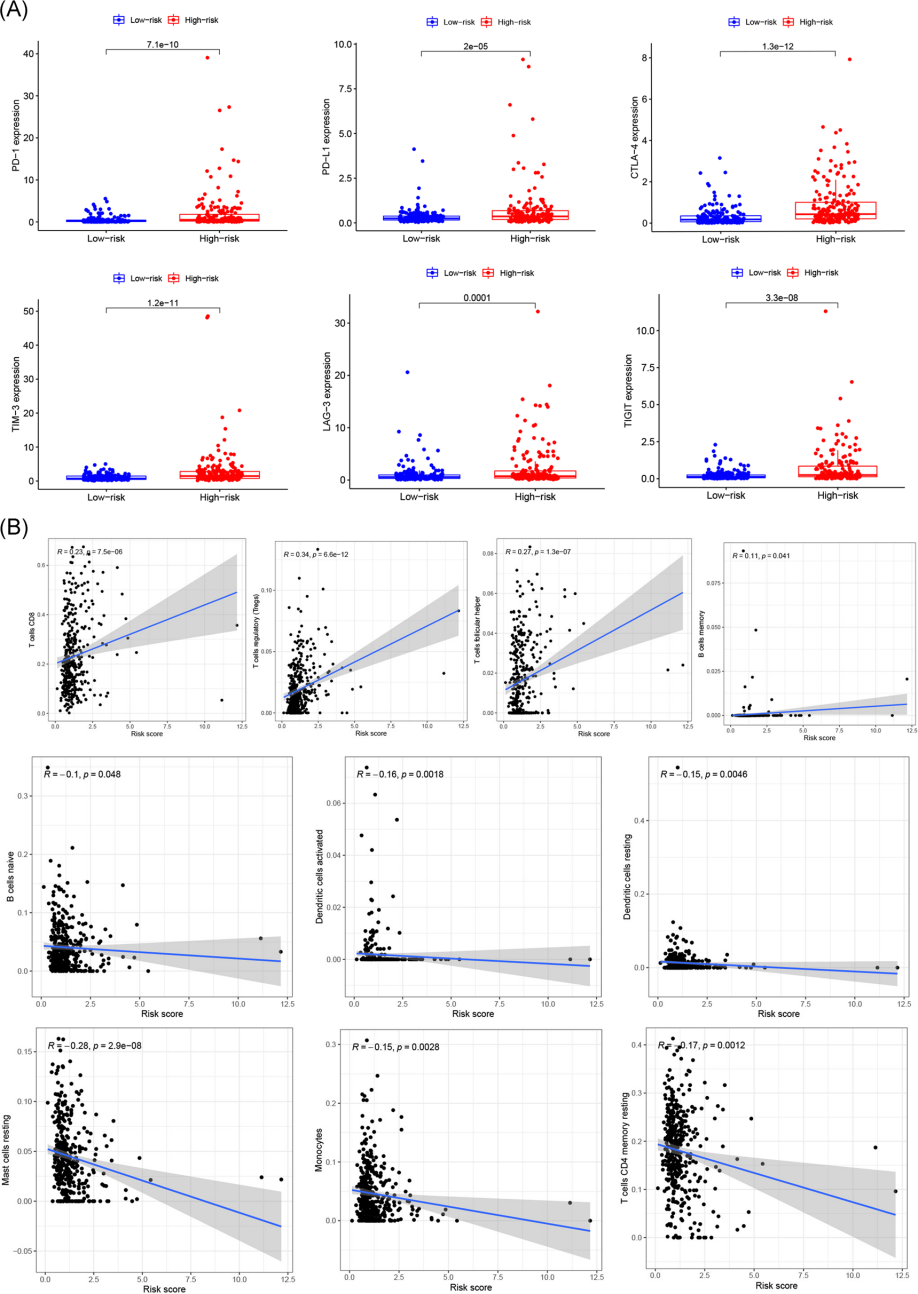
Association of prognostic risk scores with immune checkpoints and immune cell infiltration. (A) *PD‐1, PD‐L1, CTLA‐4, LAG‐3, TIM‐3*, and *TIGIT* are significantly increased in the high‐risk group. (B) The number of memory B cells, CD8 T cells, follicular helper T cells, and Tregs were positively correlated with risk scores, whereas naive B cells, activated dendritic cells, resting dendritic cells, resting mast cells, monocytes, and resting memory CD4 T cells, were negatively correlated with a risk score. Tregs, T regulatory cells

To determine whether our risk model could reflect the conditions of the immune microenvironment and to provide guidance for the immunotherapeutic response, we analyzed the differential expression of immune checkpoints among patients with different risks and the relationship between the risk score and immune cell infiltration. The expression of six immune checkpoints, that is, *PD‐1, PD‐L1, CTLA‐4, LAG‐3, TIM‐3*, and *TIGIT*, were significantly increased in the high‐risk group (Figure 8A). The number of memory B cells, CD8 T cells, follicular helper T cells, and Tregs was positively correlated with the risk score. The number of these cells increased in ccRCC tissue with an increased risk score. However, the other immune cell types, that is, naive B cells, activated dendritic cells, resting dendritic cells, resting mast cells, monocytes, and resting memory CD4 T cells, were negatively correlated with the risk score (Figure 8B).

## 4 DISCUSSION

LncRNAs have been confirmed to be abnormally expressed in a variety of malignant tumors and participate in tumor occurrence, development, invasion, and metastasis. LncRNAs play important mediating roles in cancer signal transduction pathways by interacting with proteins, RNAs, and lipids.^31^ Because lncRNAs have high organ and cell specificity, they can be found in many tissues and body fluids of patients. Some specific lncRNAs can be used as novel tumor biomarkers for tumor diagnosis, prognostic evaluation, therapeutic targets, and drug sensitivity prediction.^32^, ^33^ M6A modification is the most abundant epigenetic methylation modification in mammalian mRNAs and lncRNAs,^34^ affecting almost every process of RNA metabolism.^35^ The m6A modification of lncRNAs plays an important regulatory role in the occurrence and development of a variety of cancers. For example, the level of m6A of the lncRNA NEAT1‐1 can effectively predict the risk of death of prostate cancer patients, and high levels of m6A of NEAT1‐1 are associated with prostate cancer bone metastasis.^36^ METTL3‐mediated and METTL14‐mediated m6A modification enhances the stability of LNCAROD in head and neck squamous cell carcinoma (HNSCC) cells, an effect that is associated with the high expression of LNCAROD in HNSCC.^37^ Here, we identified seven m6A‐related lncRNAs that were significantly correlated with the prognosis of ccRCC patients and used those lncRNAs to construct a risk predictive model to predict the prognosis of patients and the immune cell infiltrating TME, which is of significance for tumor immunotherapy.

In this study, 27 prognostic m6A‐related lncRNAs were identified by analyzing gene expression data of 530 ccRCC patients in the TCGA database. Among those 27 lncRNAs, 24 high‐risk lncRNAs were overexpressed in tumor tissue, suggesting that these lncRNAs might have an oncogenic effect in the occurrence and development of ccRCC. The expression of these lncRNAs was positively correlated with the m6A “writers” *RBM15* and *METTL3. RBM15* binds to the m6A complex and recruits it to a special RNA site.^38^ *METTL3* is the first m6A methyltransferase that has been extensively studied in tumors and plays a major catalytic role in the m6A addition process.^23^ *METTL3* can promote tumorigenesis and malignant progression and is highly expressed in a variety of malignant tumors, such as bladder cancer, breast cancer, and lung cancer. Overexpression of *METTL3* is an indicator of poor patient prognosis.^39^, ^40^, ^41^ It was found that the expression of METTL3‐induced lncRNAs *ABHD11‐AS1* and *LINC00958* was upregulated in non‐small‐cell lung cancer (NSCLC) and liver cancer tissues and cells and closely associated with the poor prognosis of patients.^42^, ^43^ To investigate the biological characteristics of these m6A‐related lncRNAs associated with prognosis, we classified, via consensus clustering, ccRCC patients into two clusters based on the expression of lncRNAs. We found that patients in Cluster 2 with high expression of high‐risk lncRNAs had a poor prognosis, suggesting that m6A‐related lncRNAs can be used as biomarkers to predict the prognostic risk of ccRCC. In addition, cluster typing was closely related to the expression of immune checkpoints, stromal scores, and immune cell infiltration. In Cluster 2 patients, the stromal score was low, the number of stromal cells was low, and immune‐inhibitory cell infiltration was more prominent; *PD‐1, PD‐L1, CTLA‐4, LAG‐3*, and *TIGIT* were highly expressed and positively correlated with m6A‐related lncRNAs. These results suggest that the prognostic m6A‐related lncRNAs could be a signature for the assessment of the immune cell infiltrating TME and immunotherapeutic response.

To understand the regulatory mechanism of m6A‐related lncRNAs in ccRCC, we performed pathway and functional enrichment analysis for Clusters 1 and 2. The results showed that the signaling pathways associated with tumor occurrence and development, drug resistance, and angiogenesis, such as the mTOR signaling pathway, Notch signaling pathway, VEGF signaling pathway, and ABC transporters, were enriched to varying degrees in Cluster 2 patients with a poor prognosis. mTOR is regulated by a variety of cell signals, mainly through the phosphatidylinositol 3‐kinase/protein kinase B/mTOR signaling pathway, to regulate cell proliferation, autophagy, and apoptosis, among other regulatory functions. In many cancer types, the mTOR signaling pathway is abnormally activated and is involved in tumor formation, the regulation of immune cell differentiation, and tumor metabolism.^44^ Notch is widely expressed in many species and is highly evolutionarily conserved. It affects cell differentiation, proliferation, and apoptosis and is associated with the occurrence and development of cancers. LncRNAs can not only participate in the NOTCH signaling pathway as regulatory factors of target genes but also affect the transcription of downstream genes in the nucleus.^45^ ABC transporters are a family of energy‐dependent transport proteins located on the cell membrane. ABC transporters can mediate the unidirectional efflux of antitumor drugs and cause multidrug resistance in tumors.^46^ GO analysis indicated that m6A‐related lncRNAs were involved in biological processes such as protein methylation, microtubule‐based movement regulation, and phosphatidic acid metabolism.

Based on the above results, we suggested that the m6A‐related lncRNAs are closely associated with the prognosis and biological process of ccRCC patients. According to the LASSO regression analyses, we ultimately obtained seven m6A‐related lncRNAs (*LINC00342, AC018752.1, RPL34‐AS1, AF117829.1, AC009948.2, SNHG10*, and *AL133243.3*) that were significantly correlated with prognosis and constructed a prognosis risk model. Studies have found that the long intergenic nonprotein coding RNA 00342 (*LINC00342*) can regulate the growth, invasion, and metastasis of colorectal cancer and NSCLC cells and is closely associated with the poor prognosis of patients.^47^, ^48^ The lncRNA ribosomal protein L34 antisense RNA 1 (*RPL34‐AS1*) is localized on human chromosome 4q25 and has antitumor effects in esophageal carcinoma and papillary thyroid carcinoma. RPL34‐AS1 overexpression can inhibit tumor cell proliferation and invasion and promote apoptosis.^49^, ^50^ SNHG10 has been reported to be an oncogenic lncRNA of gastric cancer, liver cancer, osteosarcoma, and other malignancies.^51^, ^52^, ^53^ It is highly expressed in a variety of malignant tumors and is involved in the proliferation, invasion, and metastasis of tumor cells. However, some studies also reported that the high expression of SNHG10 predicts a good prognosis for NSCLC patients.^54^

Survival analysis, ROC curves, and risk curves were used to analyze the accuracy and stability of the model. The results indicated that the model can accurately distinguish high‐ and low‐risk patients and accurately predict the prognostic risk of ccRCC. The stratification of different clinical traits showed that this model could effectively predict the prognosis of patients of different ages, sex, and stages. Univariate and multivariate Cox regression analyses showed that this risk model could be used as an independent prognostic indicator for ccRCC patients. In addition, we also analyzed the relationship between the expression of the prognostic m6A‐related lncRNAs in the model and different clinicopathological characteristics. We found that the high‐risk score was associated with the progression of ccRCC. Taken together, our prognostic model is reliable and can be used to identify the risk and prognosis of ccRCC patients, information that is conducive to early intervention and treatment.

In recent years, immunotherapy targeting immune checkpoints has made substantial breakthroughs, bringing new hope to ccRCC patients. However, the complex microenvironment of tumors can mediate immune escape, leading to the failure of immunotherapy.^55^ LncRNAs are overexpressed during the development, differentiation, and activation of immune cells, such as macrophages, dendritic cells, neutrophils, T cells, B cells, and bone marrow mesenchymal stem cells.^56^ Recent studies have found that lncRNAs are involved in various processes of immune response in the TME and the promotion of tumor immunosuppression^57^ and play roles in the evaluation of immunotherapeutic response in various cancers, such as endometrial cancer and liver cancer.^58^, ^59^ Huang et al.^60^ found that the lncRNA *NKILA* alters the balance between immune activating and immunosuppressive T‐cell subsets in the TME by regulating the sensitivity of apoptosis of T‐cell subsets, resulting in tumor immune escape. Wang et al.^61^ showed that lncRNA *MALAT1* promotes the immune escape of diffuse large B‐cell lymphoma by targeting miR‐195. *MALAT1* gene knockout promotes the proliferation of CD8+ T cells and inhibits the epithelial–mesenchymal transition‐like signal transduction process through Ras‐extracellular signal‐regulated kinase signaling pathways. The lncRNA *SNHG15* promotes PD‐L1 expression through the inhibition of miR‐141 and participates in the immune escape of gastric cancer.^62^ Currently, there are limited studies that have investigated the effects of m6A‐regulated lncRNAs on the immune microenvironment of ccRCC. In this study, we found that the expression of *PD‐1, PD‐L1, CTLA‐4, LAG‐3, TIM‐3*, and *TIGIT* was upregulated in high‐risk patients, who are more likely to benefit from immunotherapy. The risk score was positively correlated with the infiltration of memory B cells, CD8 T cells, follicular helper T cells, and Tregs. The risk score was negatively correlated with the infiltration of naive B cells, activated dendritic cells, resting dendritic cells, resting mast cells, monocytes, and resting memory CD4 T cells. These results suggest that m6A‐related lncRNAs are involved in the regulation of the immune microenvironment. Pan et al.^63^ found that dendritic cells resting, dendritic cells activated, mast cells resting, mast cells activated, and eosinophils are associated with favorable prognosis in patients with ccRCC, whereas B cells memory, T cells follicular helper, and Tregs are correlated with poorer outcome.

This study has some limitations. The risk model constructed in this study was based on a public clinical database TCGA. The results were confirmed in a TCGA cohort but lacked external validation. Therefore, further validation in larger multicenter clinical patient cohorts is needed in the future. In addition, the specific regulatory mechanism of the m6A‐related lncRNAs in ccRCC requires further study.

## 5 CONCLUSION

This study systematically analyzed the prognostic value of m6A‐related lncRNAs in ccRCC patients, as well as their significance in the assessment of immune microenvironment and immunotherapeutic response. A prognostic risk model based on seven m6A‐related lncRNAs was constructed and validated. The model can predict the prognosis of patients with different clinical stratifications and the progression of ccRCC. The risk score can be used as an independent prognostic indicator for ccRCC. In addition, the m6A‐related lncRNAs were related to the expression of immune checkpoints and immune cell infiltration. Our study provides a method for the individualized risk stratification of ccRCC patients, provides a basis for further exploring the mechanisms underlying the occurrence and development of ccRCC. These m6A‐related lncRNAs could be potential targets for improving the response to immunotherapy in patients with ccRCC.

## CONFLICT OF INTERESTS

The authors declare that the research was conducted in the absence of any commercial or financial relationships that could be construed as a potential conflict of interest.

## AUTHOR CONTRIBUTIONS

Jinchang Huang and Xin Jiang conceived and designed the study; Zhenjia Fan and Shanhui Zhan downloaded and organized The Cancer Genome Atlas data; Yuqin Qiu and Xiaogang Wang performed data analysis and wrote the paper; Jinchang Huang and Xin Jiang critically revised the article for essential intellectual content and administrative support. All authors read and approved the final manuscript.

## ETHICS STATEMENT

The data of the patients in this study were obtained from the public database datasets.

## Supporting information

Supporting information.

Supporting information.

## Data Availability

The authors declare that the data supporting the findings of this study are available in The Cancer Genome Atlas database (https://portal.gdc.cancer.gov).
