# Supplementary material for: Integrated analysis on the N6‐methyladenosine‐related long noncoding RNAs prognostic signature, immune checkpoints, and immune cell infiltration in clear cell renal cell carcinoma
Source: Immun Inflamm Dis. 2021 Aug 25;9(4):1596–612. doi: 10.1002/iid3.513 (PMC8589390; doi:10.1002/iid3.513)
Supplement: Supplementary file 1 — Supporting information. [file IID3-9-1596-s001.pdf]

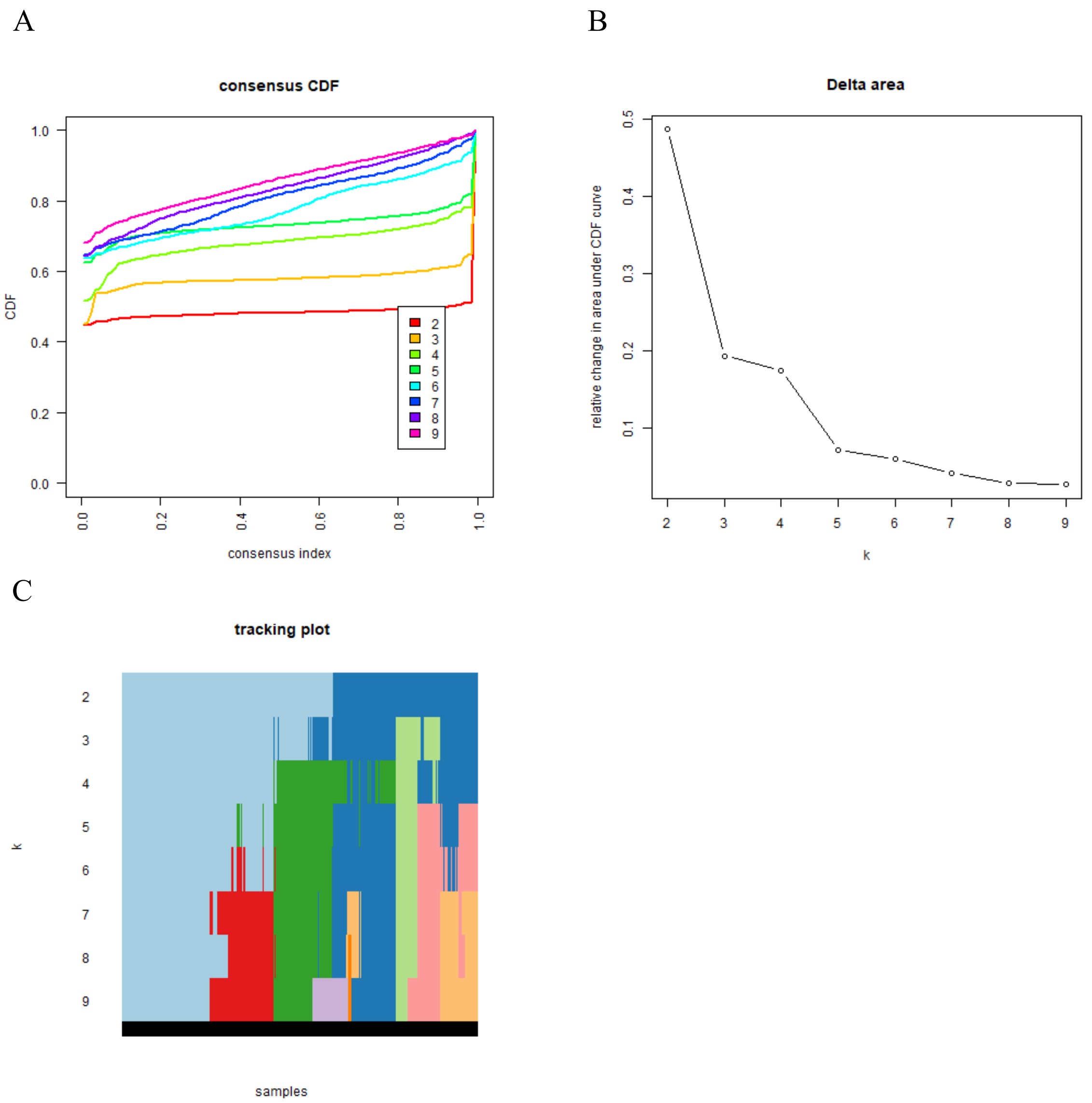

Figure S1. Consensus clusters of m6A-related lncRNAs. (A) Consensus clustering cumulative distribution function (CDF) for k=2 to 9. (B) Relative change in area under the CDF curve for k=2 to 9. (C) Tracking plot for k=2 to 9.

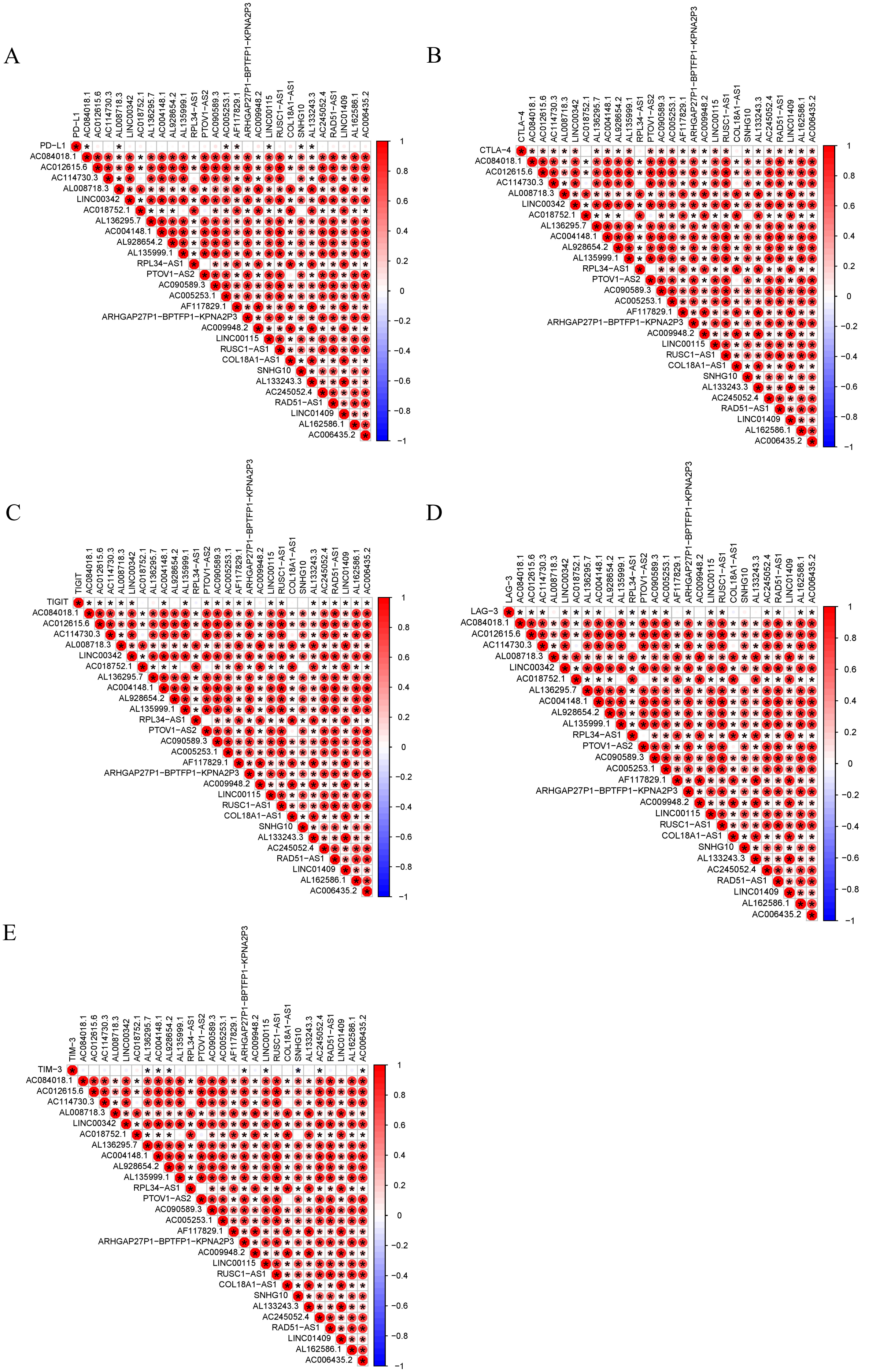

Figure S2. Correlation between the expression of immune checkpoints and m6A-related lncRNA. (A) PD-L1; (B) CTAL-4; (C) TIGIT; (D) LAG-3; (E) TIM-3. Red represents a positive correlation, while blue represents a negative correlation, with darker colors indicating greater correlation coefficients. * Indicates statistical significance between two genes, i.e., $p < 0.05$. The blank space indicate no correlation between genes.
