# Supplementary material for: Integrated analysis on the N6‐methyladenosine‐related long noncoding RNAs prognostic signature, immune checkpoints, and immune cell infiltration in clear cell renal cell carcinoma
Source: Immun Inflamm Dis. 2021 Aug 25;9(4):1596–612. doi: 10.1002/iid3.513 (PMC8589390; doi:10.1002/iid3.513)
Supplement: Supplementary file 2 — Supporting information. [file IID3-9-1596-s002.docx]

**Table S1. List of 239 m6A-related lncRNAs**

| m6A | lncRNA | cor | pvalue | Regulation |
| --- | --- | --- | --- | --- |
| METTL3 | AC084018.1 | 0.718033 | 1.46E-86 | postive |
| METTL3 | AC012615.6 | 0.717829 | 1.72E-86 | postive |
| METTL3 | AC114730.3 | 0.725256 | 4.20E-89 | postive |
| METTL3 | AL365330.1 | 0.730251 | 6.57E-91 | postive |
| METTL3 | SNHG20 | 0.743368 | 7.54E-96 | postive |
| METTL3 | AC007066.2 | 0.752302 | 2.17E-99 | postive |
| METTL3 | SEMA3F-AS1 | 0.711285 | 2.93E-84 | postive |
| METTL3 | LINC00342 | 0.70601 | 1.66E-82 | postive |
| METTL3 | AC109460.2 | 0.702718 | 1.98E-81 | postive |
| METTL3 | AL136295.7 | 0.748514 | 7.19E-98 | postive |
| METTL3 | AC004148.1 | 0.77443 | 7.51E-109 | postive |
| METTL3 | AL928654.2 | 0.802158 | 2.37E-122 | postive |
| METTL3 | AL135999.1 | 0.790441 | 2.13E-116 | postive |
| METTL3 | PTOV1-AS2 | 0.722284 | 4.77E-88 | postive |
| METTL3 | AC090589.3 | 0.727991 | 4.36E-90 | postive |
| METTL3 | AC005253.1 | 0.712712 | 9.67E-85 | postive |
| METTL3 | AC138028.4 | 0.703617 | 1.01E-81 | postive |
| METTL3 | ARHGAP27P1-BPTFP1-KPNA2P3 | 0.702332 | 2.64E-81 | postive |
| METTL3 | AC005519.1 | 0.781646 | 3.58E-112 | postive |
| METTL3 | LINC00115 | 0.705466 | 2.51E-82 | postive |
| METTL3 | RUSC1-AS1 | 0.730894 | 3.82E-91 | postive |
| METTL3 | PSMA3-AS1 | 0.79404 | 3.47E-118 | postive |
| METTL3 | AL096870.2 | 0.747602 | 1.65E-97 | postive |
| METTL3 | SNHG10 | 0.736935 | 2.18E-93 | postive |
| METTL3 | AL022328.2 | 0.723038 | 2.58E-88 | postive |
| METTL3 | AC022150.2 | 0.700759 | 8.50E-81 | postive |
| METTL3 | AC245052.4 | 0.722186 | 5.16E-88 | postive |
| METTL3 | AL139287.1 | 0.752181 | 2.43E-99 | postive |
| METTL3 | RAD51-AS1 | 0.789617 | 5.41E-116 | postive |
| METTL3 | AL162586.1 | 0.729104 | 1.72E-90 | postive |
| METTL3 | AC006435.2 | 0.727779 | 5.20E-90 | postive |
| RBM15 | AL731568.1 | 0.70731 | 6.21E-83 | postive |
| RBM15 | ADAMTSL4-AS1 | 0.77883 | 7.33E-111 | postive |
| RBM15 | AC087286.1 | 0.741885 | 2.83E-95 | postive |
| RBM15 | AL133230.2 | 0.705956 | 1.73E-82 | postive |
| RBM15 | AC084032.1 | 0.713564 | 4.97E-85 | postive |
| RBM15 | AC124312.3 | 0.7378 | 1.03E-93 | postive |
| RBM15 | AC022898.2 | 0.728374 | 3.17E-90 | postive |
| RBM15 | KANSL1L-AS1 | 0.732666 | 8.51E-92 | postive |
| RBM15 | AC080162.1 | 0.758088 | 9.16E-102 | postive |
| RBM15 | AC010285.1 | 0.729193 | 1.60E-90 | postive |
| RBM15 | AC053527.2 | 0.740717 | 7.95E-95 | postive |
| RBM15 | PCA3 | 0.723071 | 2.51E-88 | postive |
| RBM15 | CPEB2-DT | 0.73069 | 4.54E-91 | postive |
| RBM15 | AC100821.2 | 0.702181 | 2.96E-81 | postive |
| RBM15 | NFIA-AS1 | 0.701182 | 6.21E-81 | postive |
| RBM15 | AC007347.1 | 0.700302 | 1.19E-80 | postive |
| RBM15 | AC108102.1 | 0.741319 | 4.67E-95 | postive |
| RBM15 | AC087277.2 | 0.757269 | 2.01E-101 | postive |
| RBM15 | AL035658.1 | 0.707264 | 6.43E-83 | postive |
| RBM15 | AC046158.2 | 0.747547 | 1.74E-97 | postive |
| RBM15 | AC009305.1 | 0.730915 | 3.75E-91 | postive |
| RBM15 | EDRF1-AS1 | 0.723429 | 1.88E-88 | postive |
| RBM15 | AC036103.1 | 0.714809 | 1.87E-85 | postive |
| RBM15 | AC131391.1 | 0.702855 | 1.79E-81 | postive |
| RBM15 | AC012254.3 | 0.722578 | 3.75E-88 | postive |
| RBM15 | AC093732.1 | 0.75356 | 6.70E-100 | postive |
| RBM15 | AC099811.1 | 0.703422 | 1.17E-81 | postive |
| RBM15 | AL136115.1 | 0.731097 | 3.22E-91 | postive |
| RBM15 | AL365356.1 | 0.719041 | 6.52E-87 | postive |
| RBM15 | AL008718.3 | 0.72214 | 5.36E-88 | postive |
| RBM15 | LINC01473 | 0.754119 | 3.96E-100 | postive |
| RBM15 | AL590006.1 | 0.734706 | 1.49E-92 | postive |
| RBM15 | AC018752.1 | 0.734456 | 1.84E-92 | postive |
| RBM15 | ZRANB2-AS2 | 0.769133 | 1.72E-106 | postive |
| RBM15 | AF230666.1 | 0.716987 | 3.35E-86 | postive |
| RBM15 | AC004223.4 | 0.729734 | 1.01E-90 | postive |
| RBM15 | LANCL1-AS1 | 0.729475 | 1.26E-90 | postive |
| RBM15 | BACH1-IT2 | 0.72347 | 1.81E-88 | postive |
| RBM15 | AC017101.1 | 0.778526 | 1.01E-110 | postive |
| RBM15 | AC008264.2 | 0.706139 | 1.51E-82 | postive |
| RBM15 | N4BP2L2-IT2 | 0.705582 | 2.30E-82 | postive |
| RBM15 | CELF2-AS1 | 0.735777 | 5.92E-93 | postive |
| RBM15 | AC007496.1 | 0.730853 | 3.96E-91 | postive |
| RBM15 | MIRLET7A1HG | 0.731442 | 2.40E-91 | postive |
| RBM15 | AL021937.3 | 0.725496 | 3.44E-89 | postive |
| RBM15 | DIP2A-IT1 | 0.701967 | 3.47E-81 | postive |
| RBM15 | AC016747.3 | 0.726516 | 1.48E-89 | postive |
| RBM15 | AC073367.1 | 0.718306 | 1.17E-86 | postive |
| RBM15 | LINC01515 | 0.726254 | 1.84E-89 | postive |
| RBM15 | AC021739.4 | 0.705392 | 2.66E-82 | postive |
| RBM15 | AL590729.1 | 0.723203 | 2.26E-88 | postive |
| RBM15 | SLC16A12-AS1 | 0.713024 | 7.58E-85 | postive |
| RBM15 | AC093423.2 | 0.716667 | 4.32E-86 | postive |
| RBM15 | AL035071.2 | 0.70917 | 1.50E-83 | postive |
| RBM15 | AC092162.2 | 0.727351 | 7.42E-90 | postive |
| RBM15 | WASHC5-AS1 | 0.705562 | 2.34E-82 | postive |
| RBM15 | AC025031.4 | 0.725611 | 3.13E-89 | postive |
| RBM15 | AC073569.1 | 0.725934 | 2.40E-89 | postive |
| RBM15 | AC073130.2 | 0.707338 | 6.07E-83 | postive |
| RBM15 | COL18A1-AS2 | 0.755386 | 1.20E-100 | postive |
| RBM15 | SCARNA9 | 0.751843 | 3.33E-99 | postive |
| RBM15 | AC099811.5 | 0.725645 | 3.05E-89 | postive |
| RBM15 | SHANK2-AS2 | 0.712421 | 1.21E-84 | postive |
| RBM15 | AC015853.3 | 0.723608 | 1.62E-88 | postive |
| RBM15 | BACH1-IT1 | 0.707203 | 6.73E-83 | postive |
| RBM15 | AP000753.2 | 0.70334 | 1.24E-81 | postive |
| RBM15 | ITPRIP-AS1 | 0.726979 | 1.01E-89 | postive |
| RBM15 | AL162274.1 | 0.706929 | 8.29E-83 | postive |
| RBM15 | RPL34-AS1 | 0.721098 | 1.25E-87 | postive |
| RBM15 | AC079209.2 | 0.755289 | 1.32E-100 | postive |
| RBM15 | AC124283.3 | 0.719188 | 5.79E-87 | postive |
| RBM15 | KLF7-IT1 | 0.761499 | 3.39E-103 | postive |
| RBM15 | AC136424.1 | 0.730215 | 6.77E-91 | postive |
| RBM15 | LINC02863 | 0.75114 | 6.40E-99 | postive |
| RBM15 | TBL1XR1-AS1 | 0.742331 | 1.90E-95 | postive |
| RBM15 | AL157402.2 | 0.737734 | 1.09E-93 | postive |
| RBM15 | EFCAB14-AS1 | 0.740281 | 1.17E-94 | postive |
| RBM15 | ERI3-IT1 | 0.729281 | 1.48E-90 | postive |
| RBM15 | EML4-AS1 | 0.748219 | 9.42E-98 | postive |
| RBM15 | AC093864.1 | 0.742208 | 2.12E-95 | postive |
| RBM15 | AL050343.3 | 0.702715 | 1.98E-81 | postive |
| RBM15 | LYST-AS1 | 0.712397 | 1.24E-84 | postive |
| RBM15 | AP005131.4 | 0.746207 | 5.87E-97 | postive |
| RBM15 | AP003043.1 | 0.760023 | 1.42E-102 | postive |
| RBM15 | AC025423.1 | 0.75645 | 4.38E-101 | postive |
| RBM15 | AC116903.2 | 0.723928 | 1.25E-88 | postive |
| RBM15 | HCG18 | 0.752144 | 2.52E-99 | postive |
| RBM15 | AP005131.1 | 0.74229 | 1.97E-95 | postive |
| RBM15 | STARD13-AS | 0.756141 | 5.87E-101 | postive |
| RBM15 | AC073326.1 | 0.721269 | 1.08E-87 | postive |
| RBM15 | AL133342.1 | 0.718055 | 1.43E-86 | postive |
| RBM15 | AC068790.7 | 0.748667 | 6.24E-98 | postive |
| RBM15 | AC005746.1 | 0.700702 | 8.87E-81 | postive |
| RBM15 | NUTM2B-AS1 | 0.708865 | 1.89E-83 | postive |
| RBM15 | AL049869.3 | 0.720037 | 2.93E-87 | postive |
| RBM15 | AC068790.4 | 0.713313 | 6.05E-85 | postive |
| RBM15 | CFLAR-AS1 | 0.758201 | 8.22E-102 | postive |
| RBM15 | AC068533.3 | 0.70966 | 1.03E-83 | postive |
| RBM15 | AC004918.5 | 0.713828 | 4.05E-85 | postive |
| RBM15 | AC008659.1 | 0.722736 | 3.30E-88 | postive |
| RBM15 | AL157786.1 | 0.715088 | 1.50E-85 | postive |
| RBM15 | AC092839.2 | 0.721319 | 1.04E-87 | postive |
| RBM15 | AC079866.2 | 0.703958 | 7.82E-82 | postive |
| RBM15 | AF117829.1 | 0.701859 | 3.76E-81 | postive |
| RBM15 | AC007878.1 | 0.75497 | 1.78E-100 | postive |
| RBM15 | AC090948.2 | 0.748428 | 7.78E-98 | postive |
| RBM15 | AL359922.2 | 0.724861 | 5.81E-89 | postive |
| RBM15 | AL110115.1 | 0.748217 | 9.43E-98 | postive |
| RBM15 | AC007906.1 | 0.733049 | 6.14E-92 | postive |
| RBM15 | Z68871.1 | 0.747297 | 2.18E-97 | postive |
| RBM15 | AC073046.1 | 0.74703 | 2.78E-97 | postive |
| RBM15 | AL139807.1 | 0.733603 | 3.83E-92 | postive |
| RBM15 | AC068790.6 | 0.733527 | 4.09E-92 | postive |
| RBM15 | DLEU2 | 0.753024 | 1.11E-99 | postive |
| RBM15 | ARHGAP26-IT1 | 0.711171 | 3.20E-84 | postive |
| RBM15 | AL590723.1 | 0.72606 | 2.16E-89 | postive |
| RBM15 | AC022272.1 | 0.737792 | 1.03E-93 | postive |
| RBM15 | AC009948.2 | 0.708645 | 2.24E-83 | postive |
| RBM15 | AC015971.1 | 0.778973 | 6.29E-111 | postive |
| RBM15 | AL603756.1 | 0.702639 | 2.10E-81 | postive |
| RBM15 | AC008906.2 | 0.702707 | 2.00E-81 | postive |
| RBM15 | AL161725.1 | 0.70642 | 1.22E-82 | postive |
| RBM15 | AC026124.2 | 0.719801 | 3.54E-87 | postive |
| RBM15 | AC068790.2 | 0.759515 | 2.32E-102 | postive |
| RBM15 | AC087276.3 | 0.78106 | 6.73E-112 | postive |
| RBM15 | DLEU2L | 0.733459 | 4.33E-92 | postive |
| RBM15 | AC092653.1 | 0.714448 | 2.49E-85 | postive |
| RBM15 | AC005920.2 | 0.716504 | 4.92E-86 | postive |
| RBM15 | AC087286.2 | 0.787903 | 3.70E-115 | postive |
| RBM15 | AC068790.3 | 0.733611 | 3.81E-92 | postive |
| RBM15 | AC027796.5 | 0.71339 | 5.70E-85 | postive |
| RBM15 | U73169.1 | 0.71239 | 1.24E-84 | postive |
| RBM15 | AL158212.1 | 0.709294 | 1.36E-83 | postive |
| RBM15 | AC007000.4 | 0.707438 | 5.63E-83 | postive |
| RBM15 | AC096708.2 | 0.712511 | 1.13E-84 | postive |
| RBM15 | AC087749.2 | 0.710019 | 7.79E-84 | postive |
| RBM15 | AC007598.1 | 0.736204 | 4.10E-93 | postive |
| RBM15 | COL18A1-AS1 | 0.717435 | 2.35E-86 | postive |
| RBM15 | MIR29B2CHG | 0.707303 | 6.24E-83 | postive |
| RBM15 | AC008937.3 | 0.701248 | 5.92E-81 | postive |
| RBM15 | MIR17HG | 0.712698 | 9.78E-85 | postive |
| RBM15 | ETV5-AS1 | 0.728656 | 2.50E-90 | postive |
| RBM15 | AC004832.5 | 0.705585 | 2.30E-82 | postive |
| RBM15 | AC016405.1 | 0.700948 | 7.39E-81 | postive |
| RBM15 | AP005131.6 | 0.743877 | 4.79E-96 | postive |
| RBM15 | AC093690.1 | 0.73006 | 7.72E-91 | postive |
| RBM15 | AC005070.3 | 0.712781 | 9.17E-85 | postive |
| RBM15 | AC068790.5 | 0.703123 | 1.46E-81 | postive |
| RBM15 | AC098798.1 | 0.750648 | 1.01E-98 | postive |
| RBM15 | AC103739.2 | 0.71113 | 3.31E-84 | postive |
| RBM15 | AC016586.1 | 0.74222 | 2.10E-95 | postive |
| RBM15 | AC107241.1 | 0.724607 | 7.15E-89 | postive |
| RBM15 | CLMAT3 | 0.716449 | 5.13E-86 | postive |
| RBM15 | AL049840.7 | 0.722131 | 5.40E-88 | postive |
| RBM15 | BACH1-AS1 | 0.745407 | 1.21E-96 | postive |
| RBM15 | AC087286.4 | 0.751652 | 3.98E-99 | postive |
| RBM15 | AP001020.3 | 0.721681 | 7.77E-88 | postive |
| RBM15 | KCNMA1-AS3 | 0.735397 | 8.22E-93 | postive |
| RBM15 | FGF14-AS1 | 0.744366 | 3.09E-96 | postive |
| RBM15 | AL035416.1 | 0.709333 | 1.32E-83 | postive |
| RBM15 | RORA-AS1 | 0.738322 | 6.51E-94 | postive |
| RBM15 | AL355916.2 | 0.731757 | 1.84E-91 | postive |
| RBM15 | AC022726.1 | 0.726036 | 2.21E-89 | postive |
| RBM15 | AC103739.1 | 0.725331 | 3.95E-89 | postive |
| RBM15 | AC253536.3 | 0.727511 | 6.50E-90 | postive |
| RBM15 | AC090186.1 | 0.707046 | 7.58E-83 | postive |
| RBM15 | PLCB1-IT1 | 0.716573 | 4.66E-86 | postive |
| RBM15 | AP000919.2 | 0.726291 | 1.79E-89 | postive |
| RBM15 | AC092574.2 | 0.718235 | 1.24E-86 | postive |
| RBM15 | AC004492.1 | 0.701781 | 3.98E-81 | postive |
| RBM15 | AC010761.3 | 0.75656 | 3.94E-101 | postive |
| RBM15 | AL159972.1 | 0.720236 | 2.50E-87 | postive |
| RBM15 | AC004832.4 | 0.731873 | 1.67E-91 | postive |
| RBM15 | AC025043.1 | 0.710198 | 6.79E-84 | postive |
| RBM15 | CDC42-IT1 | 0.706278 | 1.36E-82 | postive |
| RBM15 | MAST4-AS1 | 0.723607 | 1.62E-88 | postive |
| RBM15 | AC007601.2 | 0.741861 | 2.89E-95 | postive |
| RBM15 | ALG13-AS1 | 0.706552 | 1.10E-82 | postive |
| RBM15 | SNHG22 | 0.735517 | 7.41E-93 | postive |
| RBM15 | PSMD6-AS2 | 0.741242 | 5.00E-95 | postive |
| RBM15 | AC090181.2 | 0.712276 | 1.36E-84 | postive |
| RBM15 | AL133243.3 | 0.727789 | 5.16E-90 | postive |
| RBM15 | LINC02605 | 0.712642 | 1.02E-84 | postive |
| RBM15 | AL158071.5 | 0.706516 | 1.13E-82 | postive |
| RBM15 | AC012409.2 | 0.731817 | 1.75E-91 | postive |
| RBM15 | LINC02157 | 0.754758 | 2.17E-100 | postive |
| RBM15 | AC008737.1 | 0.703851 | 8.48E-82 | postive |
| RBM15 | AC011825.2 | 0.70222 | 2.87E-81 | postive |
| RBM15 | AC011752.1 | 0.758447 | 6.49E-102 | postive |
| RBM15 | MADD-AS1 | 0.730428 | 5.66E-91 | postive |
| RBM15 | AL138963.1 | 0.717903 | 1.62E-86 | postive |
| RBM15 | AC007598.2 | 0.729788 | 9.70E-91 | postive |
| RBM15 | AC008937.2 | 0.728467 | 2.93E-90 | postive |
| RBM15 | AL512506.1 | 0.705479 | 2.49E-82 | postive |
| RBM15 | DHDDS-AS1 | 0.716519 | 4.86E-86 | postive |
| RBM15 | AC110296.1 | 0.730958 | 3.62E-91 | postive |
| RBM15 | AP005131.5 | 0.715364 | 1.21E-85 | postive |
| RBM15 | AP001178.2 | 0.738105 | 7.86E-94 | postive |
| RBM15 | AC087749.1 | 0.725903 | 2.46E-89 | postive |
| RBM15 | LINC01409 | 0.713144 | 6.91E-85 | postive |
| RBM15 | AC010186.3 | 0.737884 | 9.53E-94 | postive |
| RBM15 | AC116158.1 | 0.765095 | 9.86E-105 | postive |
| RBM15 | LINC00630 | 0.740317 | 1.13E-94 | postive |
| RBM15 | AC005540.1 | 0.747372 | 2.04E-97 | postive |
| RBM15 | TTN-AS1 | 0.725246 | 4.23E-89 | postive |
| RBM15 | AC078778.1 | 0.758942 | 4.03E-102 | postive |
| RBM15 | AC134349.1 | 0.767309 | 1.08E-105 | postive |
| RBM15 | USP12-AS1 | 0.727528 | 6.41E-90 | postive |

**Table S2. List of 27 m6A-related lncRNAs related to ccRCC prognosis**

| gene | HR | HR.95L | HR.95H | pvalue |
| --- | --- | --- | --- | --- |
| AC084018.1 | 1.13563 | 1.074006 | 1.200791 | 7.89E-06 |
| AC012615.6 | 1.511624 | 1.25589 | 1.819432 | 1.25E-05 |
| AC114730.3 | 1.74683 | 1.33802 | 2.280546 | 4.12E-05 |
| AL008718.3 | 2.361463 | 1.476076 | 3.777929 | 0.000338 |
| LINC00342 | 1.167971 | 1.110991 | 1.227874 | 1.17E-09 |
| AC018752.1 | 0.61498 | 0.507665 | 0.744979 | 6.73E-07 |
| AL136295.7 | 1.269107 | 1.141182 | 1.411372 | 1.10E-05 |
| AC004148.1 | 1.25713 | 1.155028 | 1.368259 | 1.19E-07 |
| AL928654.2 | 1.135413 | 1.061472 | 1.214505 | 0.000219 |
| AL135999.1 | 1.455761 | 1.249963 | 1.695444 | 1.37E-06 |
| RPL34-AS1 | 0.00044 | 9.65E-06 | 0.020024 | 7.28E-05 |
| PTOV1-AS2 | 1.13642 | 1.081737 | 1.193868 | 3.72E-07 |
| AC090589.3 | 1.347702 | 1.174701 | 1.546181 | 2.07E-05 |
| AC005253.1 | 1.460722 | 1.174566 | 1.816593 | 0.000658 |
| AF117829.1 | 1.380041 | 1.159222 | 1.642924 | 0.000294 |
| ARHGAP27P1-BPTFP1-KPNA2P3 | 1.270496 | 1.132671 | 1.425092 | 4.38E-05 |
| AC009948.2 | 2.88471 | 1.623108 | 5.126924 | 0.000305 |
| LINC00115 | 1.657452 | 1.367653 | 2.008657 | 2.56E-07 |
| RUSC1-AS1 | 1.32377 | 1.17621 | 1.489843 | 3.30E-06 |
| COL18A1-AS1 | 0.042649 | 0.007053 | 0.257889 | 0.00059 |
| SNHG10 | 1.395437 | 1.243428 | 1.566029 | 1.49E-08 |
| AL133243.3 | 2.319985 | 1.563847 | 3.441725 | 2.89E-05 |
| AC245052.4 | 2.697553 | 1.56236 | 4.657564 | 0.000369 |
| RAD51-AS1 | 1.127651 | 1.055889 | 1.20429 | 0.000342 |
| LINC01409 | 6.185952 | 2.28014 | 16.7823 | 0.000345 |
| AL162586.1 | 1.276947 | 1.160859 | 1.404643 | 4.98E-07 |
| AC006435.2 | 1.363235 | 1.192227 | 1.558773 | 5.87E-06 |

**Table S3. Clinicopathological features between train set and test set.**

|  | **train set** | **test set** | **p value** |
| --- | --- | --- | --- |
| **Total** | 266 | 264 |  |
| **Age** |  |  | 0.137 |
| ≤65 | 178 | 170 |  |
| ＞65 | 88 | 94 |  |
| **Gender** |  |  | 0.696 |
| Female | 96 | 91 |  |
| Male | 170 | 173 |  |
| **T** |  |  | 0.709 |
| T1 | 133 | 139 |  |
| T2 | 32 | 37 |  |
| T3 | 95 | 83 |  |
| T4 | 6 | 5 |  |
| **N** |  |  | 0.506 |
| N0 | 124 | 115 |  |
| N1 | 6 | 10 |  |
| Nx | 136 | 139 |  |
| **M** |  |  | 0.498 |
| M0 | 214 | 206 |  |
| M1 | 43 | 35 |  |
| Mx | 9 | 23 |  |
| **Stage** |  |  | 0.297 |
| I | 129 | 137 |  |
| II | 27 | 30 |  |
| III | 64 | 58 |  |
| IV | 46 | 36 |  |
| Unknown | 0 | 3 |  |
| **Grade** |  |  | 0.824 |
| G1 | 6 | 8 |  |
| G2 | 109 | 119 |  |
| G3 | 107 | 99 |  |
| G4 | 40 | 34 |  |
| Unknown | 4 | 4 |  |
